# Pregnancy, Viral Infection, and COVID-19

**DOI:** 10.3389/fimmu.2020.01672

**Published:** 2020-07-07

**Authors:** Ricardo Wesley Alberca, Nátalli Zanete Pereira, Luanda Mara Da Silva Oliveira, Sarah Cristina Gozzi-Silva, Maria Notomi Sato

**Affiliations:** ^1^Laboratory of Dermatology and Immunodeficiencies, LIM-56, Department of Dermatology, School of Medicine and Institute of Tropical Medicine of São Paulo, University of São Paulo, São Paulo, Brazil; ^2^Institute of Biomedical Sciences, University of São Paulo, São Paulo, Brazil

**Keywords:** COVID-19, SARS-CoV-2, pregnancy, neonatal, immunology

## Abstract

Pregnancy comprises a unique immunological condition, to allow fetal development and to protect the host from pathogenic infections. Viral infections during pregnancy can disrupt immunological tolerance and may generate deleterious effects on the fetus. Despite these possible links between pregnancy and infection-induced morbidity, it is unclear how pregnancy interferes with maternal response to some viral pathogens. In this context, the novel coronavirus (SARS-CoV-2) can induce the coronavirus diseases-2019 (COVID-19) in pregnant women. The potential risk of vertical transmission is unclear, babies born from COVID-19-positive mothers seems to have no serious clinical symptoms, the possible mechanisms are discussed, which highlights that checking the children's outcome and more research is warranted. In this review, we investigate the reports concerning viral infections and COVID-19 during pregnancy, to establish a correlation and possible implications of COVID-19 during pregnancy and neonatal's health.

## Pregnancy

Pregnancy comprises a unique immunological condition, to protect the fetus from maternal rejection, allowing adequate fetal development and protection against microorganisms (1, 2).

The maternal immune system is challenged by paternal alloantigens expressed both by the fetus and the placenta. However, through a complex range of cells and molecules, the mother does not develop a classic response to this allograft (3).

During pregnancy, fetal microquimerism occurs, where fetal cells, such as nucleated erythrocytes, trophoblastic cells, and leukocytes (3), cross the placental barrier and expose the mother to fetal alloantigens. These cells can remain in the bloodstream and maternal tissues many years after delivery (4, 5).

In comparison to the post-partum period, pregnancy increases monocytes, granulocytes, pDCs, mDCs in the blood, peaking during 2 trimesters. Simultaneously, during pregnancy occurs a reduction in CD3, CD4, and CD8 T cells in comparison with post-partum. B cells are decreased during the third trimesters. NK cells CD56 dim are reduced in the second and third trimester of pregnancy in comparison with the first trimester and post-partum period. During the second and the third trimesters, NK and CD4 T cells present a reduction in the production of IFN-γ, TNF, IL-6 cells, compared with post-partum (6) but the variability and contradictory reports are noted (7).

Maternal monocytes do not show differences in absolute numbers, however, they show some phenotypic changes including an increase in the expression of adhesion molecules (CD11a, b; CD54), and the high-affinity IgG receptor, FcγR-I (CD64) (8). The absolute number of NK cells in maternal blood increases in the first trimester of pregnancy (9).

Like lymphocytes, B cells are decreased during pregnancy and remain lower until 1 month after delivery. *In vitro*, B cells of pregnant women were less responsive, with suppression of lymphopoiesis and exclusion of autoreactive B cells (10). Despite this, vaccine response during pregnancy remains effective (11, 12).

From the 13th week of gestation, maternal peripheral blood monocytes also undergo phenotypic and functional changes. There is an increase in the ability to produce cytokines IL-1β and IL-12 and a reduction in the potential for TNF-α secretion (13). The placenta is a transient chimeric organ that develops from the uterine wall and can express different receptors and dynamically delivered microvesicles through pregnancy (14). This organ mediates hormonal, nutritional, and oxygen support to the fetus while modulating maternal's immune response (15). The placental maternal face is formed from decidual cells, with the presence of wide range of immune cells, including uterine Natural Killer (uNK), dendritic cells (DCs), and regulatory T cells (Tregs). The fetal face consists of the placental villus, which contains fetal blood vessels surrounded by fibroblasts and placental villous macrophages of fetal origin, Hofbauer cells (16, 17).

Treg cells are crucial for proper gestational development and are numerically elevated during pregnancy, in peripheral, deciduous and umbilical cord blood (18). Paternal HLA-C is a crucial molecule that can elicit allogeneic immune responses by maternal cell and aid in the development of maternal-fetal tolerance (19), also T reg may regulate CD4^+^ and CD8^+^ T lymphocyte activation through the expression of IL-10 and TGFβ (20).

Another striking feature of the maternal-fetal interface is the accumulation of NK cells, which comprise up to 70% of deciduous leukocytes in early pregnancy (21). These cells are important for the regulation of cytokines production, especially IL-10, and act in the production of angiogenic factors, chemokines, controlling the invasion of trophoblasts and availability of adequate maternal blood at the implantation site (17, 21, 22).

During pregnancy, hormonal variations can modulate immune responses, generating a reduction in the number of DCs and monocytes, and a decrease in the activation of macrophages, T, and B cells (23). To better establish the tolerogenic milieu, estrogen induces efficiently Foxp3 T regs cells (24–26).

## Viral Infection and Pregnancy

Changes in hormonal levels and immune system function generated by pregnancy may increase women's vulnerability to infections. Pregnant women show higher mortality rates and complications associated with viral infections compared to the general population (27, 28). For example, varicella disease in children is mild, but primary infections during pregnancy can progress to varicella pneumonia and death (29).

In 2009, during the H1N1 flu pandemic, an increased ratio of female to male cases was verified, in which pregnant women developed more complications, as severe acute respiratory syndrome, and higher mortality compared to the general population (30, 31). Similarly, in 1918 the pandemic Spanish flu, among 1,350 reported cases of influenza in pregnant women, 27% died as a result of the infection (32). In 1957, with the H5N1 pandemic, 50% of influenza deaths in women of reproductive age in Minnesota occurred in pregnant women (33). Although influenza viruses are restricted to maternal lungs, inflammatory cytokines can lead to fetal complications mainly preterm birth and fetus miscarriage (34, 35).

In the Ebola epidemic in 1995, 46% of infected women (out of a total of 177) were pregnant (36). Some evidence suggests that during pregnancy there is a greater risk of developing serious illnesses, spontaneous abortion, hemorrhage, and death when infected with the Ebola virus (37). Additionally, infection by the Lassa virus in pregnant women shows high levels of placental replication, and the risk of maternal-fetal mortality increases with the duration of pregnancy (38, 39).

Viruses can gain access to the decidua and placenta by ascending from the lower reproductive tract or via hematogenous transmission, viral tropism for the decidua and placenta is then dependent on viral entry receptor expression in these tissues as well as on the maternal immune response to the virus (16).

A range of viral infections in pregnancy are associated with specific placental findings, including lymphoplasmacytic villitis with associated enlargement of villi and intravillous hemosiderin deposition in the setting of maternal cytomegalovirus infection (40), as well as rare reports of intervillositis in the setting of Zika virus (41) and Dengue virus (42), among others.

Although there is little knowledge about placental findings associated with the common coronaviruses, Ng et al. reported placental pathology in seven women with SARS infection in Hong Kong (43). In three placentas delivered in the acute stage of SARS, demonstrated increased perivillous or subchorionic fibrin, while in two women who had recovered from third-trimester infection by the time of delivery, there were large zones of avascular villi, with one of the two additionally demonstrating a large villous infarct; both contained increased nucleated red blood cells in the fetal circulation. None of the seven placentas examined had any acute or chronic inflammatory processes (43).

The COVID-19 pandemic is still in its early stages, with preliminary case series of infection in pregnant women available. A study of three placentas delivered from pregnant women with SARS-CoV-2 infection, infected in their third trimester with emergency cesarean section, describe various degrees of fibrin deposition. The fibrin deposition occurred inside and around the villi with local syncytial nodule increases in all three placentas, multiple villous infarcts in one placenta, and a chorangioma in another case. All samples from three placentas were negative for the nucleic acid of SARS-CoV-2 (44).

Another study with 16 placentas from patients with SARS-CoV-2 were examined and the most significant finding is an increase in the rate of features of maternal vascular malperfusion (MVM), most prominently decidual arteriopathy including atherosis, fibrinoid necrosis, and mural hypertrophy of membrane arterioles (45). Maternal hypertensive disorders, including gestational hypertension and preeclampsia, are the major risk factors for MVM (46), although only 1 of the patients was hypertensive in this study. Notwithstanding, SARS-CoV-2 is a virus that is expected to induce inflammation, it is relevant that neither acute inflammatory pathology (AIP) nor chronic inflammatory pathology (CIP) were increased in COVID-19 patients relative to the controls. However, none of the COVID-19 patients in this study were severely ill or undergoing a cytokine storm and it may be possible that CIP could be induced in those cases of severe systemic inflammation (45).

There few knowledges about miscarriage in women with COVID-19, one case was a pregnant woman with symptomatic coronavirus disease who experienced a second-trimester miscarriage. A stillborn infant was delivered vaginally and swabs from the axillae, mouth, meconium, and fetal blood obtained within minutes of birth tested negative for SARS-CoV-2 and bacterial infection. The fetal autopsy showed no malformations, and fetal lung, liver, and thymus biopsies were negative for SARS-CoV-2. Furthermore, amniotic fluid and vaginal swabs sampled during labor tested negative for SARS-CoV-2 and bacterial infection. Placental histology demonstrated mixed inflammatory infiltrates composed of neutrophils and monocytes in the subchorial space and unspecific increased intervillous fibrin deposition (47).

During the worldwide SARS-CoV-1 (severe acute respiratory syndrome coronavirus-1) epidemic in 2003, a notable increase in mortality and morbidity was documented in pregnant patients (48). Agreeing with previous observations that the risk of viral pneumonia is significantly higher among pregnant women compared to the rest of the population (49).

In 2012, infection with the Middle East Respiratory Syndrome (MERS-CoV) coronavirus in Saudi Arabia after the isolation of a male patient who died of severe pneumonia (50, 51). Data on the effects of MERS-CoV on pregnancy are limited, whereas there is a description of stillbirth at 5 months of gestation (52). Between 2012 and 2016, the Ministry of Health of Saudi Arabia reported the occurrence of 1,308 cases of MERS-CoV infection, five of which were pregnant (53). Despite the few descriptions, the immunological changes in pregnancy may alter the susceptibility to MERS-CoV and the severity of the clinical disease (51).

In a mice model of herpes virus infection, even in the absence of herpes virus placental passage, there was a marked increase in the levels of pro-inflammatory cytokines, including IFN-γ and TNF-α, as well as changes in fetal development (30). This scenario may result from the placenta's pro-inflammatory response generated by the infection, or it may be due to other physiological changes in the mother or placenta related to the infectious process (54).

Placental cells, predominantly trophoblasts, express TLR (Toll-like receptors) and this expression varies according to the gestational age and the differentiation stage of these cells. Viral infections can disturb the fine immune regulation at the maternal-fetal interface and lead to fetal damage, even without the virus reaching it directly (55). For example, TLR-3 expressed by trophoblasts in the first trimester of pregnancy (56), mediates rapid antiviral response (57), and induces the production of cytokines, type I interferon (IFN) and type III IFN (58). TLR7 is also expressed in trophoblasts, which induces the synthesis of anti-viral cytokines and plays a role in preventing intrauterine transmission of HBV (59). However, these inflammatory responses can be associated with complications in pregnancy, such as pre-eclampsia and/or intrauterine growth deficit (1).

In general, cytokines and IFNs are important mediators in a healthy pregnancy, due to their role in the regulation of cell function, proliferation, and gene expression. However, when dysregulated, they have the potential to interrupt fetal and placental development pathways (60).

## Neonatal Immunity and Viral Infections

The World Health Organization (WHO) estimates that about 2.5 million children died within the first month of life in 2018. Every day ~7,000 newborns die, amounting to 47% of all child mortality under the age of 5 years (61). The majority of all neonatal's deaths are due to preterm birth, intrapartum-related complications (birth asphyxia or lack of breathing at birth), infections and birth defects. Regarding the highest incidence of infection observed in early-life, it is generally attributed to an immature immune system during the transitional post-natal period (62).

Innate immune cells are composed of specialized cells, such as granulocytes (e.g., neutrophil), monocytes, macrophages, DCs and innate lymphocytes. Around 5 weeks gestation, neutrophils are present in human fetal liver parenchyma (63), when compared to the adult response, neonatal neutrophils have qualitative and quantitative impairments in the response under stress conditions, including reduced chemotaxis, respiratory burst, and extracellular traps formation (64).

The cytokine profile produced by antigen-presenting cells (APCs) monocyte/macrophage and DCs in newborn differs from those produced by adults. Typically, APCs from neonates produce less pro-inflammatory cytokines like IL-1β, TNF-α, IL-12p70, and type I IFN upon stimulation on TLRs (65). Otherwise, it produces great amounts of Th17-promoting cytokines (IL-6 and IL-23) when compared with adult cells (66). Following, the importance of anti-inflammatory response in early life is highlighted through the great amount of IL-10 produced by newborn monocyte/conventional DC (cDC) compared to adults (67).

The pattern of innate cytokine response can be attributed to two mechanisms: (i) high mononuclear cell levels of intracellular cyclic adenosine monophosphate (cAMP), a secondary messenger that suppresses Th1 but enhances Th2 and anti-inflammatory cytokine production (68) and (ii) altered DNA binding capacity of transcription factors, such as IRF3 to the promoter regions of cytokine genes secondary to age-specific chromatin (69). Curiously, neonates' DCs activation with CLR agonist Dectin or macrophage-inducible C-type lectin (Mincle), simultaneously with TLR7/8 potently drives caspase-1 and NF-kB activation and Th1-supporting cytokine production (including IL-12p70), overcoming the age-specific epigenetic barrier in early life for IRF3 function and leading to a Th-1 phenotype (70, 71). On 14 weeks of gestation, mature fetal αβ T lymphocytes can be detected. During the second and third trimesters of gestation, the repertoire of fetal T cell receptors diversifies (72). Generally, neonates have a limited Th1 profile response to some vaccines and pathogens, agreeing with a lower capacity of CD4 T cells to produce IFN-γ and of APCs to produce Th1-skewing cytokines (73). Although there are some situations where the responsiveness of the Th1 profile is efficient, for example, neonates and infants develop adult-like Th1 responses to BCG or pertussis vaccines, and a fetus can develop Th1 responses in congenital CMV infection (74–76).

Recent studies suggested that the early life immune system could present advantages for the elicitation of broadly neutralizing antibodies (bnAbs), a response highly desired for an HIV vaccine. In fact, HIV-infected children develop bnAbs responses earlier and more frequently than infected adults (77).

Congenital and perinatally acquired viral infections do occur and may lead to major disabilities in infancy and childhood, the main causes can be attributed to pathogens like *Toxoplasma gondii*, rubella virus, cytomegalovirus (CMV), herpes viruses, syphilis, and Zika virus (78). While congenital rubella virus syndrome is no longer seen in countries with compulsory immunization against this virus, an outbreak of Zika virus (ZIKV) recently occurred in Brazil resulting in the ZIKV syndrome, with brain lesions comparable to, but more severe than congenital CMV infection (79).

Neonates display an immature immune response, the first exposition to an environmental stimulus can shape the lung's immune response (80). Furthermore, there is a predominant type 2 immune response in the lungs (81), these characteristics make infants susceptible to respiratory viral infections, a common cause of infant's death (82). RSV is an important cause of lower respiratory tract illness in infants globally and is responsible for one-third of deaths due to lower respiratory tract infections in children <1 year of age (83).

Pregnant women are considered at high risk for severe influenza disease, for this reason, influenza vaccination has been recommended for pregnant women and introduced into immunization programs (84). Influenza vaccination is safe and protective on preterm birth (PTB) and low birth weight (LBW) (85). One of the benefits of maternal immunization has also been shown to extend to neonates through the transfer of maternal antibodies, providing passive immunization against the influenza virus (86).

On the severe 2009 pandemic H1N1 influenza illness, some studies suggested an association between severe H1N1 disease, preterm birth, and fetal death; however, these limited data do not permit firm conclusions (35).

SARS-CoV-1 infected ~100 pregnant women during the pandemic (87), causing a high lethality and miscarriage rate (88), but no neonatal infection has been reported (88). In 2017, Cynthia Maxwell postulated possible intensive care and procedures to properly manage maternal and neonatal SARS-CoV-1 infections (89).

Vertical transmission of MERS has not been documented. In a case report by Alserehi et al., a mother was diagnosed with MERS, treated and a cesarean section was performed to deliver a healthy preterm baby with 32 weeks of gestation (52). Hon et al. described 14 children with MERS, that presented persistent fever and cough, after treatment no fatal case was reported. All children in this report obtained the infection via adult-to-children transmission, and no children-to-children transmission was reported (90). Iqbal et al. reported a case of spontaneous vaginal delivery in COVID-19-positive pregnant, with no signs of neonatal infection up to 7-days post-partum (91). Nevertheless, it is important to highlight contact precautions were made in this report to prevent post-partum transmission.

## Immune Response and COVID-19

In late 2019, a respiratory infectious disease began to be investigated in Wuhan, China (92). At first, contagion occurred through contact with some infected animals but, soon there were the first reports of human-to-human transmission (93), The virus was identified as belonging to the *coronaviridae* family and was designated SARS-CoV-2 (severe acute respiratory syndrome coronavirus-2) (94). Like other members from this viral family, MERS and SARS-CoV-1, the new coronavirus causes a respiratory disease, named COVID-19 (coronavirus disease−2019) (95).

Although very similar, SARS-CoV-1 and SARS-CoV-2 impacted the world differently. SARS-CoV-1 emerged in 2002 and killed almost 800 people in 26 countries (96) and, even without a vaccine, it was taken preventive actions as patient isolation. The new coronavirus has killed more than 480,000 people in just 6 months and has spread to 5 continent (97).

SARS-CoV-2 shares genetic similarities between SARS-CoV-1 and MERS, 79 and 50%, respectively (98). SARS-CoV-2 is an enveloped single-stranded RNA virus and has a genome of ~30,000 nucleotides that encode structural and accessory proteins—the largest known viral RNA genome (99).

SARS-CoV-1 and SARS-CoV-2 enter the host's cells via the ACE2 receptor (angiotensin-converting enzyme 2) (100). In the lung, the most affected organ among those infected, the main target is the type 2 alveolar cell (101). The ACE2 receptor is also expressed in cells from kidneys, esophagus, heart (102). Moreover, a small percentage of monocytes and macrophages express the ACE2 receptor (94, 99). Thus, there may be another alternative receptor or infectious pathway, such as antibody-dependent enhancement (ADE). However, unlike other coronaviruses, limited to respiratory disorders, SARS-CoV-2 caused multiple organ failure. Furthermore, this receptor is more expressed in the elderly, which associated with immunosenescence and other comorbidities common among the elderly may justify the high lethality rate in this age group (103).

The viral load peaks occur during the first week of infection and then gradually decrease over the next few days. In addition, the viral load is correlated with the patient's age. IgG and IgM antibodies start to increase 10 days after disease and most patients are seroconverted in the first 20 days (104). Moreover, *in vitro* assays, has shown that the serum from SARS-CoV-2-infected patients were able to neutralize the virus (101). Thereby, the humoral response can be another antiviral strategy via plasma transfer (105). In SARS-CoV-1 and MERS, as a viral escape mechanism, the virus can suppress IFN type I response, either by cytosolic sensors of ubiquitination, inhibiting nuclear factors translocation or decreasing STAT1 phosphorylation (106).

Neutrophils, C-reactive protein and several cytokines (as IL-6, TNF, IL-10) are increased in COVID-19, and this elevation is correlated with disease severity and death (97). In serious illness, the same protein levels were detected and inflammatory cytokines increase is correlated with T CD4^+^ and T CD8^+^ lymphocytes decrease and lower IFNγ production. B-lymphocytes do not appear to be affected by the disease, regardless of severity (92, 103, 107).

These characteristics observed in patients indicate that a COVID-19 can be mediated by an intense inflammatory process that follows the disease severity. As with SARS-CoV-1 and MERS, this increase in cytokine levels—known as a cytokine storm—can be involved with the pathogenesis of the disease (92).

To defend itself against an aggressive agent (such as infection, trauma, acute inflammation, among others) the body produces an exaggerated response to localize and then eliminate the damage. This response is known as the Systemic Inflammatory Response Syndrome (SIRS) or, if the source infection sepsis (108), this process leads to the release of acute-phase proteins and endocrine, hematological and immunological changes, among them, the cytokine storm can lead to tissue damage and even death (109).

Cytokine storm is produced, mainly, by highly activated macrophages and can cause lung damage and start viral sepsis (110). This inflammation leads to other complications, such as acute respiratory distress syndrome (ARDS) and respiratory and cardiac failure (48, 111). Studies in mice infected with SARS-CoV-1, also demonstrate the cytokine storm dampening adaptive immunity (112).

Other factors may also influence the susceptibility for COVID-19 infected persons, and some gene polymorphisms, well-documented for other viral infections (113).

## COVID-19: Maternal and Neonatal Immunity

At the moment no vaccine or specific treatments are available for disease control of the SARS-CoV-2. In pregnancy, pneumonia infections may trigger an increased mortality risk to the mother and fetus (114), which can also lead to complications as preterm birth and small for gestational age (115).

Placental syncytiotrophoblast cells express the ACE2 receptor and this receptor is highly expressed in the first months of pregnancy. Associated with placental immaturity, the early ACE2 expression can make the first trimester the most likely period for SARS-CoV-2-infection (14). A serine protease, TMPRSS2, is also required for viral entry (100, 116) and there is still no consensus about placenta expression. Some studies report low, but present, mRNA expression in human placentas (117), others describe that expression is not detectable (118). The association of TMPRSS2 and ACE2 expression, in the first months of pregnancy, would make this phase more susceptible to SARS-CoV-2-infection.

Blood tests in pregnant women revealed regular COVID-19 markers, such as lymphopenia, neutrophilia, and elevated C-reactive protein level in pregnant women (119, 120). Some reports also verified an increase in ALT, AST, and D-dimer (120–122). An important report verified that 3 mothers developed anemia and dyspnea, which could potentially be a risk factor during C-section labor (123).

Chen and collaborators, verified alteration in calcium and albumin levels in the blood of pregnant women with SARS-CoV-2 infection (124), which could potentially increase the severity in COVID-19 (125). Furthermore, in a recent report involving maternal death in consequence to COVID-19, 2 cases reported a low number of platelets, which is associated with an increase in mortality by COVID-19 (126, 127).

It is still under investigation the effects of SARS-CoV-2-infection in the maternal-fetal context (Table 1).

**Table 1:** Effect of SARS-CoV-2 infection on pregnant women.

**Pregnancy semester during infection**	**Maternal diagnoses**	**Mother antibodies**	**Children infected**	**Children antibodies**	**Infant complications due to maternal COVID-19**	**Maternal treatment previous to delivery (as described by paper)**	**References**
N/A	RT-PCR	Mixed, 2 mothers with high IgM and 3 with high IgG	No	Yes, mixed results. Two newborns with IgM/IgG, and three with IgG. One newborn with very low levels of IgM and IgG.	Elevated IL-6 in all infants. None of the infants presented symptoms.	N/A	(128)
N/A	RT-PCR	N/A	Yes	N/A	Lymphopenia, deranged liver function tests, and elevated creatine kinase level. After 36 h post-partum tested positive for SARS-CoV-2	N/A	(119)
3°	RT-PCR	N/A	No	N/A	No	Lopinavir 200 mg and Ritonavir 50 mg (each 2 × /day), methylprednisolone (40 mg 1 × /day)	(129)
3°	RT-PCR	N/A	Yes, but vertical transmission could not be confirmed	N/A	No	N/A	(130)
3°	RT-PCR	Yes, IgM and IgG	No	Yes, IgM and IgG	Lymphopenia, neutrophilia, elevated aspartate aminotransferase (AST), total bilirubin, Creatine kinase, lactate dehydrogenase, IL-6, IL-10	Antiviral, antibiotic, corticosteroid, and oxygen therapies	(120)
3°	RT-PCR	N/A	No	N/A	No	Antibiotics (Gentamicin, Metronidazole and Cephazolin)	(131)
N/A	RT-PCR	N/A	No	N/A	N/A	N/A	(132)
3°	RT-PCR	N/A	No	N/A	Neutrophilia, 2 newborns with elevated AST	N/A	(133)
3°	RT-PCR	N/A	No	N/A	N/A	3 were treated with oral oseltamivir 1 treated with oral oseltamivir and nebulized inhaled interferon 6 non-treated	(121)
N/A	RT-PCR	N/A	No	N/A	2 newborns presented skin rashes after birth	N/A	(123)
3°	RT-PCR	N/A	No	N/A	No	N/A	(124)
N/A	RT-PCR	N/A	Yes, 3 from a 33 newborn cohort	N/A	3 newborns presented pneumonia	N/A	(122)
2°	RT-PCR	N/A	No	N/A	CRP increased and developed lymphopenia on day 5	Antibiotic and corticosteroids	(134)
1°	RT-PCR	N/A	N/A	N/A	No	No COVID-19 treatment	(135)
N/A	RT-PCR	N/A	Yes, 1 from a 3 newborn cohort, but vertical transmission could not be confirmed	N/A	2 tested positive for post-partum infection, 1 died, 1 developed neutrophilia, lymphopenia, and elevated lactate dehydrogenase	N/A	(136)
2°	RT-PCR	N/A	No, but the placenta was positive for SARS-CoV-2	N/A	One stillbirth	Acetaminophen	(47)
2° and 3°	RT-PCR	N/A	No vertical transmission, with one newborn acquired SARS-CoV-2 post-natally	N/A	One stillbirth	Oseltamivir 75 mg (2 × /day for 5 days); hydroxychloroquine sulfate 400 mg or chloroquine sulfate 1,000 mg (single dose) Lopinavir/ritonavir 400/100 mg and ribavirin 1,200 mg (2 × /day each for 5 days) Enoxaparin 40 mg (1 × /day) or heparin 5,000 units (2 × /day)	(137)

Some reports describe that symptomatic infected-mothers did not transmit the virus during pregnancy. In a case report of seven cases, showed that three babies were tested to SARS-CoV-2 and only 1 was positive 36 h post-partum (138). On the other hand, another report shows increase in inflammatory cytokines and virus-specific IgM levels in newborns, from infected-mothers, 2 h after birth (120), and in another report, newborns presented virus-specific IgM and IgG, but no SARS-CoV-2-infection (Table 1) (128). This lead to the possibility of the activation of the maternal immune system by SARS-CoV-2 may have some implication of the offspring's health and immune system development.

Although the number of pregnant women with COVID-19 studies is limited, there is no conclusive report of vertical transmission (Table 1) (129, 139). A recent case report, was described two cases of rashes and one with facial ulcerations (123).

Another important factor, besides the immune activation, the maternal usage of antiviral drugs can also permanently affect the offspring's immune response (140), as there is no current standard protocol of treatment regarding the usage of antibiotics or antivirals (Table 1) (115).

Only a fraction of patients infected with SARS-CoV-2 develops severe respiratory disorders, it is unknown whether the pregnant could be more susceptible to pulmonary diseases. COVID-19 can progress to a severe lung inflammation that can progress to life-threatening illness at the severe stage (141). This inflammatory process is associated with high plasma levels of cytokines, as cytokines storm, including IL-2, IL-7, IL-10, G-CSF, IP-10, MCP-1, MIP-1A, and TNFα (92).

This might play an important role in pregnancy as IL-2 has been implicated to be upregulated in pre-eclampsia (142) and miscarriage (143) and IL-7/IL-7R signaling pathway in fetal miscarriage (144), due to the upregulation in the ratio of Th17/Treg cells (145).

Another relevant aspect is the possible implication of polymorphisms in COVID-19 diseases, as is well-documented for other viral infections (114). Also, cytokines polymorphisms, such as TNF-α 308G/A (rs1800629) polymorphism is associated with recurrent miscarriage (146).

In fact, TNF-α and TNF-α receptor play an important role in the development of the fetus, being present in the ovary, endometrium, placenta, and fetus, and in the amniotic fluid in different concentration (147). This increase in TNF-α during pregnancy may implicate in different health outcomes depending on the gestational period (148), leading to tissue necrosis in the placenta and hypoxia (149). Interestingly, an acute increase of this cytokine during pregnancy in animals may cause abortion (7).

Moreover, alteration in the health status of the mother during pregnancy can have long-term effects on the offspring's health (150). Inflammatory processes during pregnancy can also impact women's health, as the increase in TNF-a during pregnancy can also lead to impaired insulin sensitivity (151) and gestational diabetes mellitus (152).

In animal models, inflammation during pregnancy has been shown to alterations in the behavior (153, 154) fetal brain development (155–157), metabolic disturbance (158, 159), and shape offspring's immune response to antigens and infections (160, 161).

The physiological response, as stress and the control of temperature, during the infection may present a long-term effect in pregnant women with COVID-19. The increase in stress-related hormones can also affect the offspring's immune system (162) and fever during pregnancy increase the chances of neural disorders in the children (163).

Moreover, an increase in anti-inflammatory cytokine IL-10 in COVID-19 mothers is probably a regulatory mechanism crucial to regulate the inflammation (164) and pregnancy maintenance (165).

Even though no vertical transmission for COVID-19 has been reported until now, several reports of early-life infections have been described with very low death rates (98, 119). Reports with recommendations to the treatment of pregnant women with COVID-19 (166) and for neonates with COVID-19 have been published (167, 168). Another possible route for SARS-CoV-2 is oral transmission by fecal samples (169), and via breastfeeding from a SARS-CoV-2 infected mother. Regarding breastfeeding, a small study found no evidence of COVID-19 in breast milk, of six patients (139). However, the primary concern is whether an infected mother can transmit the virus through respiratory droplets during breastfeeding.

Other viruses in the past have also caused concern in pregnant women. The Zika virus has been linked to several cases of microcephaly in newborns during an epidemic in 2015 in Brazil (170). The infection had a high point in the first trimester of pregnancy, where there were more favorable conditions for its entry and replication in placental cells. In the case of SARS-CoV-2, it has not yet possible due to the time of infection occurs in the world, to observe the consequences of infection in the first-trimester pregnancy. Taking into account the early pregnancy, the placental tissue immaturity together with the up-regulation of ACE2 expression in placental cells, perhaps the more susceptible period for SARS-CoV-2 infection is around the first trimester of pregnancy.

It is important to highlight that after the 2009 influenza pandemic there have been reports of reduced cytokine response to bacterial infections. This leads to the hypothesis that COVID-19 can lead to impairments of the immune response to other pathogens and vaccines in the future.

Future investigations are needed to identify the possible implications of SARS-CoV-2/COVID-19 in pregnancy, the possible infection of the placenta in the first trimester of pregnancy and implications of the cytokine storm to the neonatal's health.

## Author Contributions

RA and MS: write, conception, and review. NP, LO, and SG-S: write and review. All authors contributed to the article and approved the submitted version.

## Conflict of Interest

The authors declare that the research was conducted in the absence of any commercial or financial relationships that could be construed as a potential conflict of interest.
